# Linkage mapping of putative regulator genes of barley grain development characterized by expression profiling

**DOI:** 10.1186/1471-2229-9-4

**Published:** 2009-01-09

**Authors:** Christof Pietsch, Nese Sreenivasulu, Ulrich Wobus, Marion S Röder

**Affiliations:** 1 Leibniz Institute of Plant Genetics and Crop Plant Research (IPK), 06466 Gatersleben, Germany

## Abstract

**Background:**

Barley (*Hordeum vulgare *L.) seed development is a highly regulated process with fine-tuned interaction of various tissues controlling distinct physiological events during prestorage, storage and dessication phase. As potential regulators involved within this process we studied 172 transcription factors and 204 kinases for their expression behaviour and anchored a subset of them to the barley linkage map to promote marker-assisted studies on barley grains.

**Results:**

By a hierachical clustering of the expression profiles of 376 potential regulatory genes expressed in 37 different tissues, we found 50 regulators preferentially expressed in one of the three grain tissue fractions pericarp, endosperm and embryo during seed development. In addition, 27 regulators found to be expressed during both seed development and germination and 32 additional regulators are characteristically expressed in multiple tissues undergoing cell differentiation events during barley plant ontogeny. Another 96 regulators were, beside in the developing seed, ubiquitously expressed among all tissues of germinating seedlings as well as in reproductive tissues. SNP-marker development for those regulators resulted in anchoring 61 markers on the genetic linkage map of barley and the chromosomal assignment of another 12 loci by using wheat-barley addition lines. The SNP frequency ranged from 0.5 to 1.0 SNP/kb in the parents of the various mapping populations and was 2.3 SNP/kb over all eight lines tested. Exploration of macrosynteny to rice revealed that the chromosomal orders of the mapped putative regulatory factors were predominantly conserved during evolution.

**Conclusion:**

We identified expression patterns of major transcription factors and signaling related genes expressed during barley ontogeny and further assigned possible functions based on likely orthologs functionally well characterized in model plant species. The combined linkage map and reference expression map of regulators defined in the present study offers the possibility of further directed research of the functional role of regulators during seed development in barley.

## Background

Barley is an important crop but also a model plant for temperate cereals because of its diploid nature and rich genetics. Extensive genomics resources have been developed, and a number of important genes were already isolated by map-based cloning [1-4]. Since the grain is the agriculturally most important part of the plant many studies have been carried out on barley seed development and germination [recently reviewed in [5]]. However, we still know little about the genes determining important traits and the regulatory networks underlying developmental processes. Prime candidates for members of signal transduction chains and regulatory networks are transcription factors (TF), kinases and other experimentally verified regulators. Different approaches have been used to gain insight into the relationship between genotype and phenotype in mutant studies and the analysis of transgenic plants. One additional promising approach is the more recently developed concept of Genetical Genomics, which combines gene expression studies with genetic linkage analysis [6]. Differences in transcript levels are used to map the responsible chromosal regions thus eventually allowing the identification of the causative gene(s). In species whose genomes have not yet been sequenced such as barley, the feasibility of this strategy depends on the number of genetically mapped genes. Based on our long-term dedication to understand seed development and seed storage product accumulation as well as to study agronomical traits by extensively using marker-based techniques we set out to first define and characterize a set of genes with known or putative regulatory functions during grain development. In a second step SNP marker were developed for as many as possible of those genes and used to anchor them on the barley linkage map. Thus we provide new data on regulatory genes and their putative joint action based on cluster analysis of expression data (the 'guilty of association' concept) and place part of them on the barley linkage map to promote marker-assisted studies in barley especially on the biology of barley grains.

## Results and Discussion

### Expression map of selected regulatory genes

**Figure 1:**
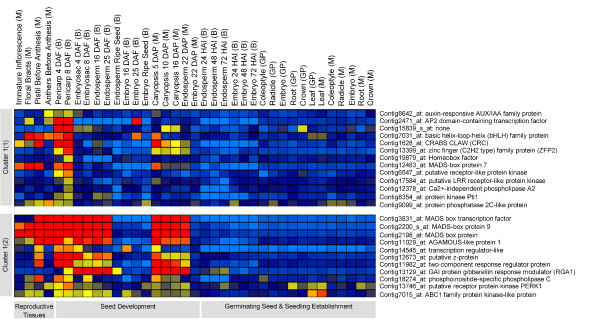
**Cluster group of genes preferentially expressed in pericarp [cluster 1(1)] and both in pericarp and endosperm fractions [cluster 1(2)]**. Fine clustering using K-means of individual clade of hierachical output resulted in identifying cluster groups. Expression values are given in logarithmic scale (base 2): red-high expression; yellow-moderate expression; blue-low expression. Each individual gene is represented as horizontal row and developmental stages are described in vertical columns. Overall, gene expression data obtained from 3 cultivars are shown: cultivar 'Barke' (B), 'Morex' (M), 'Golden Promise' (GP). Gene expression data covering independent replications are shown in Additional file 3.

**Figure 2:**
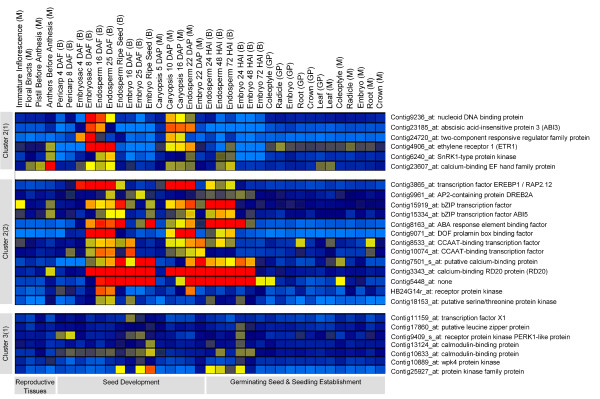
**Cluster group of genes preferentially expressed in endosperm [cluster 2(1), 2(2)] and embryo [cluster 3(1)]**. For abbreviations see legend of Figure 1.

**Figure 3:**
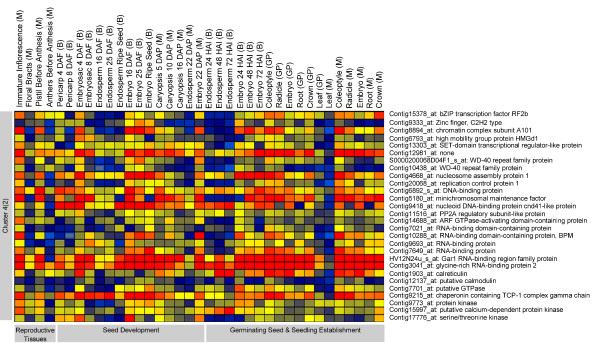
**Cluster group of genes expressed in cell differentiating tissues across barley plant ontogeny**. For abbreviations see legend of Figure 1.

To define a set of barley genes putatively involved in regulating preferentially grain development sequences from cDNA libraries of developing seeds [7] were used in similarity searches against rice and Arabidopsis databases in search for homologs of regulatory genes. From the total set, those transcription factors (TF) and signalling related genes were selected, which were shown to be exclusively expressed during seed development or alternatively characterized by a spatio-temporal pattern of gene expression, which is characteristic for both seed development and across plant ontogeny [8]. In a first step 376 regulatory sequences (172 TFs and 204 signalling related genes, mostly kinases) were selected based on their homology to known regulatory genes in other model species (Additional file 1) and on identified homologous sequences from the Affymetrix barley1 GeneChip. These genes were further characterized. Expression patterns were extracted and normalized from 95 published experiments concerning samples from both endosperm and embryo fractions of developing and imbibed seeds of cultivar Barke [9] as well as selected stages of seed development, coleoptile, radicle, root, crown and leaf from germinating seeds and immature inflorescence, floral bracts, pistil and anthers from reproductive tissues of cultivars Morex and Golden Promise [10]. A hierarchical clustering method identified major clusters with defined co-expressed regulatory genes that are associated either with specific tissues during seed development or found to be expressed in both developing seeds as well as during other stages of plant ontogeny (Additional file 2). Individual clades of the hierarchical tree were subjected to K-mean clustering to get more refined sub clusters. As a result, 50 regulatory genes were found to be preferentially expressed in one of the three studied seed tissues (pericarp, endosperm and embryo) during seed development. They are represented in cluster groups 1, 2 and 3, respectively (Figures 1 and 2). A total of 32 regulators are characteristically expressed in multiple tissues undergoing cell differentiation events during seed development, also during the shift from germination to seedling establishment as well during the transition from vegetative to generative development (cluster group 4, Figure 3; Additional file 3). In addition 27 regulators were found to be expressed during both seed development and germination (cluster 5, Additional file 3).

The first cluster contains two subclusters 1(1) and 1(2). Subcluster 1(1) contains 13 regulatory genes that are preferentially activated in pericarp during seed development as well as partly in reproductive tissues (Figure 1, Additional file 3). Examples are members of the following gene families: an auxin-responsive AUX/IAA gene (Contig8642_at), AP2 (Contig2471_at), bHLH (Contig7031_at), C2H2 Zincfinger (Contig13399_at), Homeobox (Contig19879_at), a well characterized CRABS CLAW gene (Contig1628_at) from the YABBY family and TF MADS 7 (Contig12483_at), which is homologous to the Arabidopsis *SEPALLATA *gene *AGL2*. Whereas in Arabidopsis CRABS CLAW and AGL2 members were shown to be required for carpel development [11,12], the homologous genes in barley are expressed in the pericarp during early seed developmen, as well as in the pistil. Similarly, MADS box gene transcripts, known to be involved in floral transition [13], are here found to be abundantly expressed not only in reproductive organs such as pistil and anther but also in pericarp and endosperm fractions during the onset of seed development and not in the embryo (see Contig3831, Contig2200_s_at, Contig2198_at and Contig11029_at in subcluster 1(2) (Figure 1 and Additional file 3). This indicates an unexplored distinct role of MADS box TFs in specific tissues during grain development. Similar transcript profiles were observed among signaling related genes: a two-component response regulator (Contig11982_at), the receptor protein kinase PERK1 (Contig13746_at), the ABC1 family protein kinase (Contig7015_at) and the gibberellin response modulator RGA1 (Contig13129_at).

Regulatory genes expressed preferentially during seed maturation in the endosperm are represented in subcluster 2(1). We especially note preferential expression of the well-known regulators ABI3/VP1 (Contig23185_at) and SNF1 (Contig6240_at) in endosperm during the maturation phase but not in any other tissue during plant ontogeny (Figure 2, Additional file 3). Also preferentially expressed during seed maturation in endosperm tissue and to some extent in embryo (subcluster 2(2)) were the ABA response element binding factors ABF3 (Contig15919_at, Contig8163_at) and ABI5 (Contig15334_at). Since in a recent study we found ABRE elements in the Snf1 kinase promoter region [8] it seems likely that ABA plays a role in triggering endosperm maturation events and that the hormone acts through Snf1 mediated by ABA via ABF3/ABI5 and ABI3. Probably by the same way ABA is involved in controlling starch biosynthesis during endosperm maturation [8]. Also present in subcluster 2(2) is the barley prolamin binding factor transcript (Contig9071). The respective protein is a member of the DOF family of TFs known to activate storage protein hordein B transcription during the main endosperm storage period [14]. Coexpressed with the prolamin binding factor in cluster 2(2) are two members of the CCAAT TF family (Contig8533_at, Contig10074_at). This family contains the well characterized LEC1 TF of Arabidopsis, a major seed maturation control factor of the Arabidopsis HAP2 family [15]. Taken together, most of the maturation control genes discovered in Arabidopsis were also found to be preferentially expressed during maturation events in barley. Along with these gene sets we also noticed calcium signaling related genes possibly participating in a genetic framework for seed maturation (Additional file 3), which needs further investigation.

Subcluster 1 of cluster 3 encodes 7 regulators such as transcription factor X1 (Contig11159_at), a putative leucine zipper protein (Contig17860_at), calmodulin-binding proteins (Contig13124_at, Contig10633_at), a receptor protein kinase PERK1-like protein (Contig9409_s_at), a wpk4 protein kinase (Contig10889_at) and an unclassified protein kinase (Contig25927_at) all expressed preferentially in the embryo during seed maturation (Figure 2).

The fourth cluster contains 32 regulators found to be expressed in pericarp, endosperm and embryo during the cell differentiation events in the developing seed. Its expression is reduced during the peak of storage phase as well in the mature seeds of the endosperm (Figure 3, cluster 4). Interestingly, gene expression patterns of these regulators are found to be expressed throughout embryo development. They are also expressed in the immature inflorescence, the pistil as well as in early germinating seedling tissues such as crown and coleoptile (Figure 3, cluster 4) where cell differentiation occurs. This cluster is enriched with a nucleosome/chromatin assembly factor (Contig8894_at, Contig4668_at), a SET-domain transcriptional regulator-like protein (Contig13303_at) and WD-40 proteins (S0000200068D04F1_s_at, Contig10438_at). WD-40 members belong to chromatin complex subunits of the SNF2 (Contig8894_at) family. The suppression of its homologues in Arabidopsis results in small cotyledonary embryos with limited cell expansion [16]. We furthermore observe the expression of two members of MCM genes (Contig5180_at, Contig20068_at), which are homologous to PROLIFERA, a protein required for DNA replication during seed development [17] as well as HMG family members (Contig6793_at) known to promote meristematic activities during plant development [18,19]. G-protein signalling components are also expressed.

Interestingly, 96 regulators which were found to be expressed in the developing seed were also ubiquitously expressed in all tissues of germinating seedlings as well as in reproductive tissues. Examples are AP2-EREBP, bZIP, HMG and MYB TF family members and calcium, G-protein, receptor kinases and serine/threonine protein kinase family members representing signaling components (Additional file 3).

### SNP-marker development and anchoring to the linkage map of barley

Based on the analyzed expression profiles an initial list of 172 transcription factors and 204 kinases was derived and the sequences used for SNP detection, marker development and anchoring on the barley linkage map.

Of the 376 primer pairs designed, 153 (40%) yielded a single copy-PCR product and 137 of them were sequenced from both sides for each parental line amounting to a maximal read depth of fourteen reads resulting in an average consensus length (phred quality setting ≥ 40) of 427 bp amounting to 59.78 kb sequenced in total. The remaining sixteen loci showed an indel-polymorphism or a microsatellite polymorphism between the parental lines at least in one of the four mapping populations on agarose gels. For 64 sequenced loci we detected polymorphisms between the parental lines in at least one out of four mapping populations (Additional file 4). These mapping populations comprised the two doubled haploid standard populations Steptoe/Morex (SM) and Oregon Wolf barley (OWB) [20,21], as well as two BC3 advanced backcross populations Brenda/HS213 (BHS213) and Brenda/HS584 (BHS584) which were used for QTL detection elsewhere [22,23]. Due to their population structure the advanced backcross populations are not useful for mapping of previously unassigned markers, but were only used for genotyping of markers mapped in other mapping populations.

We detected 135 SNPs in 64 sequenced polymorphic PCR products resulting in a SNP-density of 2.3 SNP per kb (Table 1). Seventy-three loci did not show a sequence polymorphism between the seven barley varieties. All detected SNPs were dinucleotide polymorphisms except the locus GBS3024 that showed a trinucleotide SNP. With respect to SNPs between the parental lines of the mapping populations the Oregon Wolfe barleys showed 62 SNP in 37 loci or genes, respectively. The number of polymorphic loci and the total number of SNPs in the Oregon Wolfe barley was more than twice as found for the Brenda/HS584 population (Table 1). Remarkably, the lowest numbers of polymorphic loci were found between the parental lines of the advanced backcross populations comprising the elite variety 'Brenda' and two *Hordeum spontaneum *accessions. This low number of polymorphic loci entails SNP-densities of 0.6 and 0.5 SNPs per kb in HS213 and HS584 versus 'Brenda', compared to a SNP density of 1.0 and 0.7 for the Oregon Wolfe barley and the Steptoe/Morex populations (Table 1). In contrast, we found a higher number of SNPs within polymorphic genes in the Brenda/HS213 and Brenda/HS584 population of 1.9 and 2.2 per gene, whereas the SNP density in Oregon Wolfe barley and the Steptoe/Morex population amounted to 1.6 SNP and 1.4 SNP per locus, respectively. The detected percentage of 22% polymorphic loci for the Steptoe/Morex population was below the value of 36% reported for the same population in [24]. A low polymorphism rate of 15% was also reported for microsatellite markers in the Brenda/HS213 population [22], while 46% of 400 tested microsatellite markers were polymorphic in the Brenda/HS584 population [23].

**Table 1:** Descriptive statistics of SNP frequency

Mapping population	Number of polymorphic loci	Number of SNPs	Mean number of SNPs per locus	SNP density [kb]
OWB	37	62	1.6	1.0
SM	29	42	1.4	0.7
BHS213	18	33	1.9	0.6
BHS584	12	28	2.2	0.5
total	64	135	2.3	2.3

Twenty-eight out of 64 SNPs, for which markers have been developed, are located in exons, whereof 16 SNPs could be assigned to UTRs (Additional file 4). The representative examples includes UTR regions with assigned SNP-sites belonged, for instance, to a well known DOF transcription factor (Contig9071_at), an activator of prolamin gene transcription expressed preferentially in endosperm during storage phase (Additional file 3, Figure 1). The other SNP-sites assigned to specific UTR-motifs includes marker GBS3014 (Contig 12673_at) and GBS3060 (Contig 15839_s_at), which were located in an IRES (internal ribosome entry site) motif that is supposed to be located in 5' UTRs [25]. The internal mRNA ribosome binding is a mechanism of translation initiation alternative to the conventional 5'-cap mechanism. The IRES motifs are 79 and 97 bp in size, respectively. Both clones clustered in the expression analysis in the first cluster group with specific expression in pericarp during seed development and partly in reproductive tissues (Additional file 3, Figure 1). The number of exonic SNPs involving transitions exceeds the number of transversions (χ^2 ^9.8, n = 44, p ≤ 0.01), whereas the ratio of transitions vs. transversions appeared to be balanced at intronic SNP-sites (χ^2 ^0.42, n = 22, p = 0.5).

Altogether, we developed 80 marker assays for 76 transcription factors and kinases showing a polymorphism at least in one out of four mapping populations (Additional file 5). Information about primer sequences, expected fragment lengths as well as the Crest IDs for the cluster-sequences that have been used as template sequence for primer design  can be found in additional files 1 and 5.

In total, we mapped 61 markers onto the SM- and OWB-maps that have been used for the construction of the barley consensus map in [24] (Additional files 6 and 7). Thirty-eight markers have been placed onto the OWB-framework map and 23 markers onto the SM-framework map. Eight markers could not be assigned to a barley linkage group. The marker locations appeared to be randomly distributed over the barley genome, although a slight accumulation is seen on chromosome 5H (Additional file 7). In addition to the mapping of polymorphic loci, we assigned the chromosomal location for 12 loci with the aid of wheat-barley chromosomal addition lines [26]. Altogether, 73 markers were allocated to the barley chromosomes of which 48 markers are based on genes which were found specifically expressed in developing seeds or expressed in developing seeds as well as in other tissues (Additional file 3). Another 11 markers could be genotyped on the advanced backcross populations Brenda/HS213 and Brenda/HS584. These two populations have been used in the past for QTL-analysis of agronomic important traits including grain-related traits, such as thousand-grain weight [22,23]. In the future we want to extend our studies and include transcriptome-based e-QTLs of grain development.

### Synteny to rice

**Figure 4:**
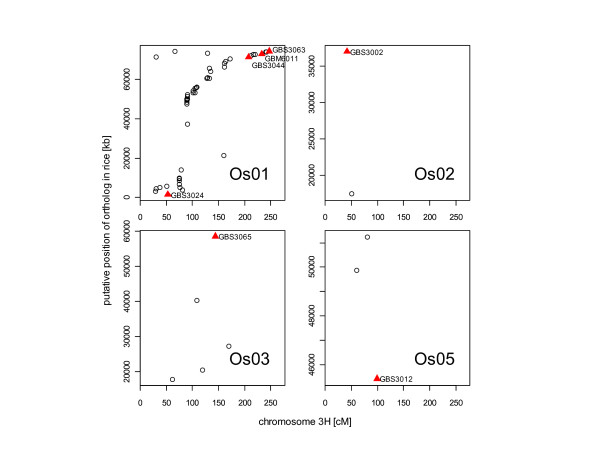
**Relationship between Oregon Wolfe Barley markers on chromosome 3H with putative orthologs in rice**. Relationship between barley markers on chromosome 3H to their putative orthologs in rice based on BlastN (E≤-10) for developed marker (triangles) and publicly available marker (dots) published by [24].

There are extensive syntenic relationships between barley and rice chromosomes that are well documented [24,27-29]. For instance, 475 out of 1032 EST-based markers in the consensus map of [24] mapped to known co-linear linkage groups in rice. To assess the synteny of the developed markers to the rice genome we performed a BlastN-analyis of the marker sequences to the rice genome in order to detect putative orthologs in rice. Then, we analysed the publicly available information on map position and BlastN-analysis of the consensus map published by [24] and counted the markers of the barley consensus map, whose best BlastN-hit mapped to the same rice chromosome as for the developed markers. For 49 mapped markers and 8 markers assigned to chromosomes in wheat barley addition lines we found a significant BlastN-hit (E ≤ 1e-10) in rice, whereas for 18 markers we did not find a putative ortholog in rice (Additional file 6). We tested the accumulation of BlastN-hits of developed and published barley markers [24] on specific rice chromosomes with a χ^2^-test assuming n markers on the linkage group divided by the number of rice-chromosomes under the Null-hypothesis. Based on this test, fourty-one developed markers obey macro co-linear relationships between barley and rice (p = 0.01). Some highlighting examples include ethylene signal transduction ETR1 gene GBS3000 (Contig4906_at); members of the AP2/EREBP family GBS3032 (Contig8357_at), GBS3030 (Contig6636_at), GBS3031 (Contig3914_s_at), GBM6020 (Contig6526_at); and other signaling genes. Figure 4 shows an example, where the developed markers GBS3024, GBS3032 GBS3044, GBS3062, GBS3063 and GBM6011 mapped on the barley chromosome 3H show their putative ortholog in rice on chromosome Os01 as the majority of published markers, whereas no syntenic relationship can be observed for the markers GBS3002, GBS3012 and GBS3065 with orthologous sites on Os02, Os05 and Os03. Only three markers (GBS3036, GBS3038, GBS3051) did not yield a significant χ^2 ^statistics but showed a syntenic relationship in a smaller region between barley and rice. Additionally, we detected for the marker GBS3046 a syntenic relationship to rice for the second best BlastN-hit. This observation may result from different evolutionary fates of gene duplications occurring in rice after the divergence of the Triticeae and Oryzeae. For the remaining twelve markers we did not find an indication for a syntenic relationship based on marker statistics. They were predominantly located on chromosome 5H but were also found on chromosome 1H, 2H, 3H and 7H.

## Conclusion

We identified expression patterns of major transcription factor genes and signaling related genes expressed during barley ontogeny and selected putative regulators expressed preferentially in pericarp, endosperm and embryo during seed development and germination based on clustering and possible functions. For a total of 76 candidate genes SNP and indel-marker development was conducted and allowed the anchoring of 61 markers on the genetic linkage map of barley. Exploration of the macrosynteny to rice revealed that the chromosomal order of the putative regulatory factors is in most cases conserved during evolution. The combined linkage map and reference expression map of regulators defined in the present study offers the possibility for further directed research on the nature and role of regulators during seed development and germination in barley.

## Methods

### Gene annotation and selection of regulators

Based on ESTs generated from four cDNA libraries of developing barley seed tissues [7] a 12K unigene set was developed for barley [8]. The consensi sequences were used to identify putative regulators based on homology searches to already defined regulatory sequences in Arabidopsis and rice with a cut off e value of 10^-10 ^(see [8]). Based on the available transcriptome data produced by using the 12K seed array we identified 376 putative regulators expressed during barley seed development by identifying differentially expressed genes with respect to developmental time and/or tissue (pericarp, endosperm and embryo fractions) during fertilization to late seed maturation [8]. These defined regulators showed confined spatio-temporal gene expression patterns inherent to pericarp, endosperm and embryo fractions between fertilization and the late maturation stages. In the present study we selected these regulators to define (a) genome wide gene expression patterns using the Affymetrix Barley1 Genechip covering different stages of barley life cycle and (b) for the prediction of SNP sites.

### Affymetrix Barley1 GeneChip analysis

First, we identified highly homologous sequences between the selected 376 putative regulators (see above) and sequences represented on the Affymetrix Barley1 GeneChip based on best BLAST results with an arbitrary e value of 10^-20 ^or better. To assess gene expression patterns of these selected regulators during barley plant development we downloaded cel files from publicly available reference experiments [10] covering various tissues (coleoptile, radicle, root, crown and leaf) and stages of seed germination, from reproductive tissues (immature inflorescence, floral bracts, pistil and anthers) as well as a detailed transcriptome dataset of developing and imbibed seeds from both endosperm and embryo fractions [9]. Expression data were normalized using the MAS5 method and for each tissue covering temporal development a linear model was fitted using limma (according to [30]). Eventually the normalized expression data were transformed to a logarithmic scale (log2) and subjected first to hierarchical clustering using Genesis software [31] to find the major expression patterns. To gain further clarity we subjected individual clades of hierarical output to K-mean clustering using Genesis software [31]. Eventually the coexpressed subsets of genes were extracted from selected cluster groups and heat maps prepared using Cluster 3.0 software. In order to validate the statistical relevance for cluster group1, 2 and 3 showing preferential expression in pericarp, endosperm and embryo tissues, respectively we calculated differentially expression ratio between seed tissues versus non-seed tissue such as leaf and root by applying a significance threshold of 0.01 in combination with Benjamini-Hochberg false-discovery rate and confirmed its seed specificity.

### Plant material, DNA extraction

Seven barley varieties have been selected for polymorphisms screening that belong to four doubled haploid mapping populations: Steptoe/Morex (SM) [20], Oregon Wolfe Barley Dominant/Oregon Wolfe Recessive (OWB) [21], Brenda/*H. spontaneum *accession HS213 (BHS213) [22] and Brenda/*H. spontaneum *HS584 (BHS584) [23]. Additionally the variety Barke was included in the screening for polymorphism. The SM population comprised 78 individuals, the OWB population 89 individuals. The BHS213 and BHS584 are BC3-DH populations which were used for QTL detection, however, are not suitable for the placing of previously unmapped markers. As an additional reference the variety Barke was included which had been used for creating expression analysis data [8]. Additional segregation data were obtained from GrainGenes 2.0  for the SM population, and from the Oregon State University (OSU) Barley Project website  for OWB, Total genomic DNA was extracted from ~4–6 g young leaf material using the protocol described in [32].

### Primer design, PCR, sequencing, discovery of polymorphisms

Unigene sequences from the crop expressed sequence tag database CR-EST (clustering project g03)  served as template for primer design using Primer3 [33]. Primers were designed for 58–60°C annealing temperature and ideally bracket a 400–600 bp fragment depending on the EST singelton or cluster size, respectively. PCR was performed in a total reaction volume of 35 μl with 50–100 ng genomic DNA, reaction buffer containing 1.5 mM MgCl_2_, 0.2 mM dNTPs and 10 μM each primer and 1 U *Taq *DNA polymerase. Primers were supplied by Metabion (Martinsried, Germany). Amplification was carried out in GeneAmp PCR System 9700 thermocycler. The thermocycling profile consisted of a 3 min denaturation step at 94°C DNA followed by 45 cycles of 1 min at 94°C, 1 min at 60°C, 2 min at 72°C, followed by a final extension step by 7 min at 72°C.

A volume of 10 μl of the PCR products of the eight parental varieties were separated on 1.5% agarose gel to check for a single copy PCR product (2 h at 100 V). Primer pairs showing a fragment length polymorphism between the parental lines of a mapping population were directly used for genotyping. Single copy PCR products were purified with the MinElute™ 96 UF PCR Purification kit (Qiagen, Hilden, Germany) according to the manufacturer's instructions. Afterwards, PCR products were subjected to sequencing from both sides using the same primers as for PCR amplification. Cycle sequencing was performed with the BigDye Terminator v3.1 ready reaction cycle sequencing kit on an ABI 3730x1 sequencer (Applied Biosystems).

ABI chromatograms were trimmed with the Phred quality setting of 40 (1 error in 10.000 bp) [34]. The Phred-Phrap-Consed suite  was used to assemble trimmed scf-traces with given settings using the PhredPhrap Perl script distributed with Consed [35]. SNPs were detected with PolyPhred [36] and by visual inspection of Phrap assemblies. We considered only homozygous SNP-sites in the wild barley accessions HS584 and HS213. Consensus sequences have been exported only for high-quality sequences with a Phred quality setting of 40.

### SNP assay design and genotyping

SNPs were predominantly genotyped using the pyrosequencing technique [37,38] and assays have been developed with the Pyrosequencing™ Assay Design Software Version 1.0.6 (Biotage AB, Uppsala, Sweden). For single DNA-strand preparation we used Streptavidin Sepharose™ High Performance, from Amersham Biosciences (Freiburg, Germany). Primers for pyrosequencing were supplied by Metabion (Martinsried, Germany), and PSQTM HS 96 A SNP reagents by Biotage AB (Uppsala, Sweden).

Seven controls have been included to assess the occurrence of non-specific signals in pyrosequencing due to primer-dimers: 1. sequencing primer with annealing buffer (AB), 2. biotinylated-primer with AB, 3. sequencing primer, biotinylated-primer with AB, due to annealing of the biotinylated-primers, 4. biotinylated-primer with PCR-product an AB, due to 3'-end loops, 5. PCR-product with AB to control for PCR background-signals, 6. negative PCR-control with sequencing primer and AB, 7. negative PCR-control with biotinylated primer and AB.

The SNP genotyping has been performed on the PSQ HS 96 A System (Biotage AB, Uppsala, Sweden). The PCR was carried out in a total volume of 35 μl with 50–100 ng genomic DNA, reaction buffer containing 1.5 mM MgCl_2_, 0.2 mM dNTPs and 10 μM of biotinylated and unlabeled primer, and 1 U *Taq *DNA polymerase. The temperature profile comprised a 3 min DNA denaturation step at 94°C followed by 45 cycles of 15 s at 94°C, 30 s at 58°C, 15 s at 72°C, followed by an extension step of 4 min at 72°C. PCR products were checked on 2% agarose gel in order to separate small fragments from primer clouds. A volume of 12 μl of biotinylated PCR product was used for the pyrosequencing reaction. Further information on template preparation and the pyrosequencing protocol can be found in [39].

SNPs for which the pyrosequencing assay design turned out to be difficult were subjected to CAPS marker design with the SNP2CAPS program [40] or to dCAPS development with the SNP Cutter program [41]. The digestion reaction was carried out in a total reaction volume of 25 μl with 20 μl PCR product, 1 U restriction enzyme and a final concentration of 1× digestion buffer. Samples were digested at the appropriate temperature according to the manufacturer's instruction for 3 h. Subsequently the reaction was inactivated by applying 80°C for 20 min. Electrophoresis was carried out with 10 μl of digested PCR product on a 1.5% agarose gel for 2 h with 100 V.

### Mapping of developed molecular markers

Newly developed molecular markers for transcription factors and kinases were mapped onto the Steptoe × Morex and Oregon Wolfe barley maps that have been used for the construction of the consensus map in [24] using the 'distribute' command in Map Manager QTX 0.3  with a linkage criterion of p ≤ 0.01. Additionally, we mapped publicly available marker onto the framework-maps and used these marker for the assignment of the BIN position of the developed marker. Informations for the BIN-membership of the publically available markers are derived from [42] and from  (last update 08/11/06). The linkage maps with information on the barley map bin assignment and the putative rice ortholog of the developed markers were drawn with MapChart [43].

Loci which yielded single copy PCR-products but did not show a polymorphism within the tested mapping populations were assigned to specific chromosomes or specific arms of chromosomes using a set of twelve wheat-barley chromosome addition lines (cv 'Chinese Spring')/*H. vulgare *(cv 'Betzes') covering 2HL, 2HS, 3HL, 3HS, 4HL, 4HS, 5H, 5HL, 6H, 6HL, 7H and 7HS [26].

In order to analyze the syntenic relationship for the developed markers between barley and rice, we mapped the gDNA sequences for SNP markers and EST cluster sequences for indel markers to the rice genome by BlastN (E ≤ 1e-10) using the osa1 database version 4.0 (December 15, 2006) at .

### Gene annotation, characterisation of SNP sites

We used several tools in order to ascertain the location of SNP-sites in the intron-, exon- or UTR-regions. Firstly, we queried gDNA-sequences for SNP containing sequences and cDNA sequences for loci showing an indel-polymorphism in the annotation system RiceGAAS [44] employing the gene-prediction programs RiceHMM [45], MZEF [46] or GENSCAN [47] using the default settings. When the analysis did not yield a hit in the rice-related sequences, we performed a spliced alignment with gDNA and cDNA of EST cluster sequences with Genseqer  using a rice specific splice-site model [48]. Sequences of SNP locations that have not been assigned to internal exons in the RiceHMM analysis and were not found in the corresponding EST-cluster sequence were screened for functional sequence patterns located in 5' or 3' UTRs using UTRscan [49].

## Authors' contributions

CP carried out SNP analysis, development and mapping of Pyrosequencing and CAPS markers, statistical analysis and drafted part of the manuscript. NS selected all genes involved in the analysis, performed the cluster analysis, biological interpretation and drafted gene expression part of the manuscript. UW conceived the study and drafted the manuscript. MRS conceived the study, coordinated the project, sequenced part of the genes and drafted the manuscript.

## Supplementary Material

Additional file 1 **Total list of investigated genes**. List of 172 transcription factors (TF) and 204 kinases including information about Blast N hits. Click here for file

Additional file 2 **Identification of major cluster groups of co-expressed regulatory genes expressed in a tissue and development-specific manner during barley plant ontogeny**. Hierarchical clustering of all 376 genes resulted in major clades shown as tree on left side. Expression values are given in logarithmically scale (base 2): red for high expression; yellow for moderate expression; blue for low expression. Each individual gene is represented as horizontal row and developmental stages are described in vertical columns. For further details see Figure 1. Click here for file

Additional file 3 **List of 205 regulator genes expressed in developing seed.** Identification of 3 cluster groups (clusters 1–3) containing 109 regulators preferentially expressed in one of the three tissues maternal pericarp or filial endosperm and embryo during seed development; identification of another 7 cluster groups (clusters 4–10) with further 96 regulators which were expressed in developing seed and were ubiquitously expressed among all tissues of germinating seedlings as well among reproductive tissues. Click here for file

Additional file 4 **SNP site detection.** List of SNPs found in the mapping populations Steptoe/Morex (SM), Oregon Wolf Barley (OWB), Brenda/HS213 (BHS213) and Brenda/HS584 (BHS584). Click here for file

Additional file 5 **Information for genotyping assays or amplification in wheat barley chromosomal addition lines**. Primer information for Pyrosequencing, CAPS and indel assays. Click here for file

Additional file 6 **Markers mapped in OWB- or SM-populations or in wheat-barley chromosomal addition lines (WB) with reference to the rice genome**. Chromosomal position in the linkage maps of barley and most significant Blast hits on the genomic sequence of rice. The macrosyntenic relationships of barley – rice are compared to other ESTs mapped in both species. Click here for file

Additional file 7 **Barley linkage map with novel integrated markers**. The markers were integrated in the framework of the Oregon Wolf Barley (OWB) mapping population and the Steptoe × Morex (SM) mapping population. Syntenic loci in the rice physical map are indicated. Click here for file
