# Supplementary figures and images for: Linkage mapping of putative regulator genes of barley grain development characterized by expression profiling
Source: BMC Plant Biol. 2009 Jan 9;9:4. doi: 10.1186/1471-2229-9-4 (PMC2648977; doi:10.1186/1471-2229-9-4)

Reproductive  
tissues

Seed development

Germinating seed and seedling establishment

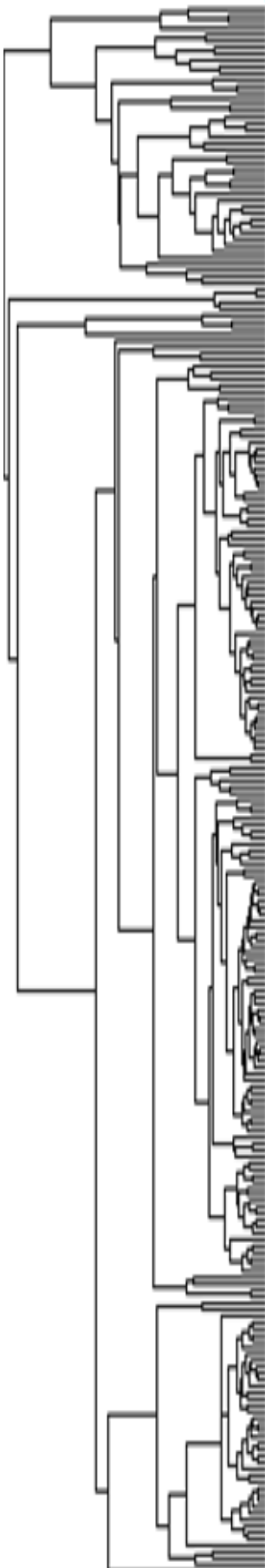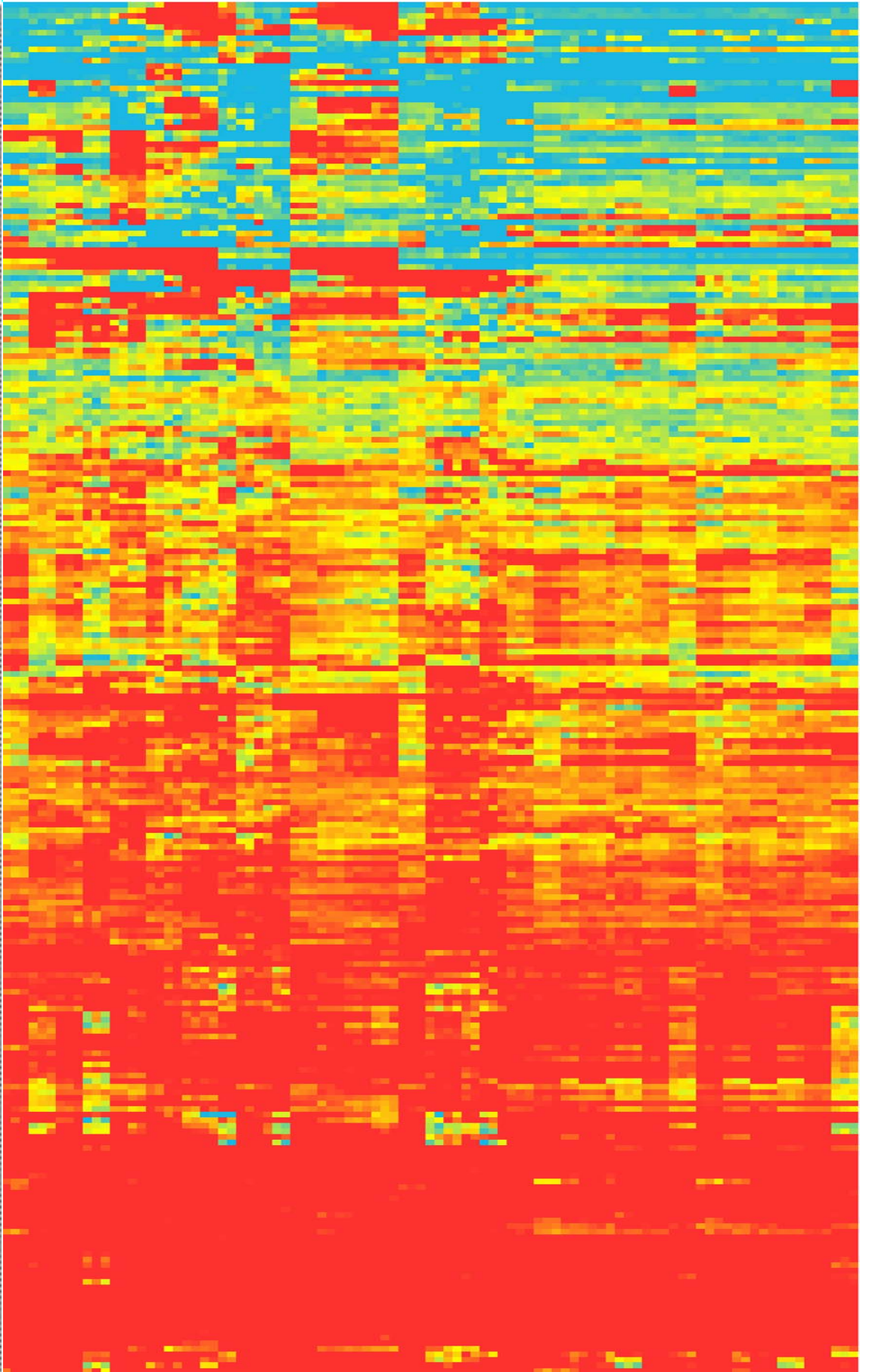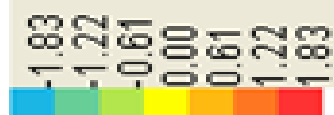

Supplement: Additional file 2 — Identification of major cluster groups of co-expressed regulatory genes expressed in a tissue and development-specific manner during barley plant ontogeny. Hierarchical clustering of all 376 genes resulted in major clades shown as tree on left side. Expression values are given in logarithmically scale (base 2): red for high expression; yellow for moderate expression; blue for low expression. Each individual gene is represented as horizontal row and developmental stages are described in vertical columns. For further details see Figure 1. [file 1471-2229-9-4-S2.pdf]
