# Supplementary material for: Linkage mapping of putative regulator genes of barley grain development characterized by expression profiling
Source: BMC Plant Biol. 2009 Jan 9;9:4. doi: 10.1186/1471-2229-9-4 (PMC2648977; doi:10.1186/1471-2229-9-4)
Supplement: Additional file 7 — Barley linkage map with novel integrated markers. The markers were integrated in the framework of the Oregon Wolf Barley (OWB) mapping population and the Steptoe × Morex (SM) mapping population. Syntenic loci in the rice physical map are indicated. [file 1471-2229-9-4-S7.pdf]

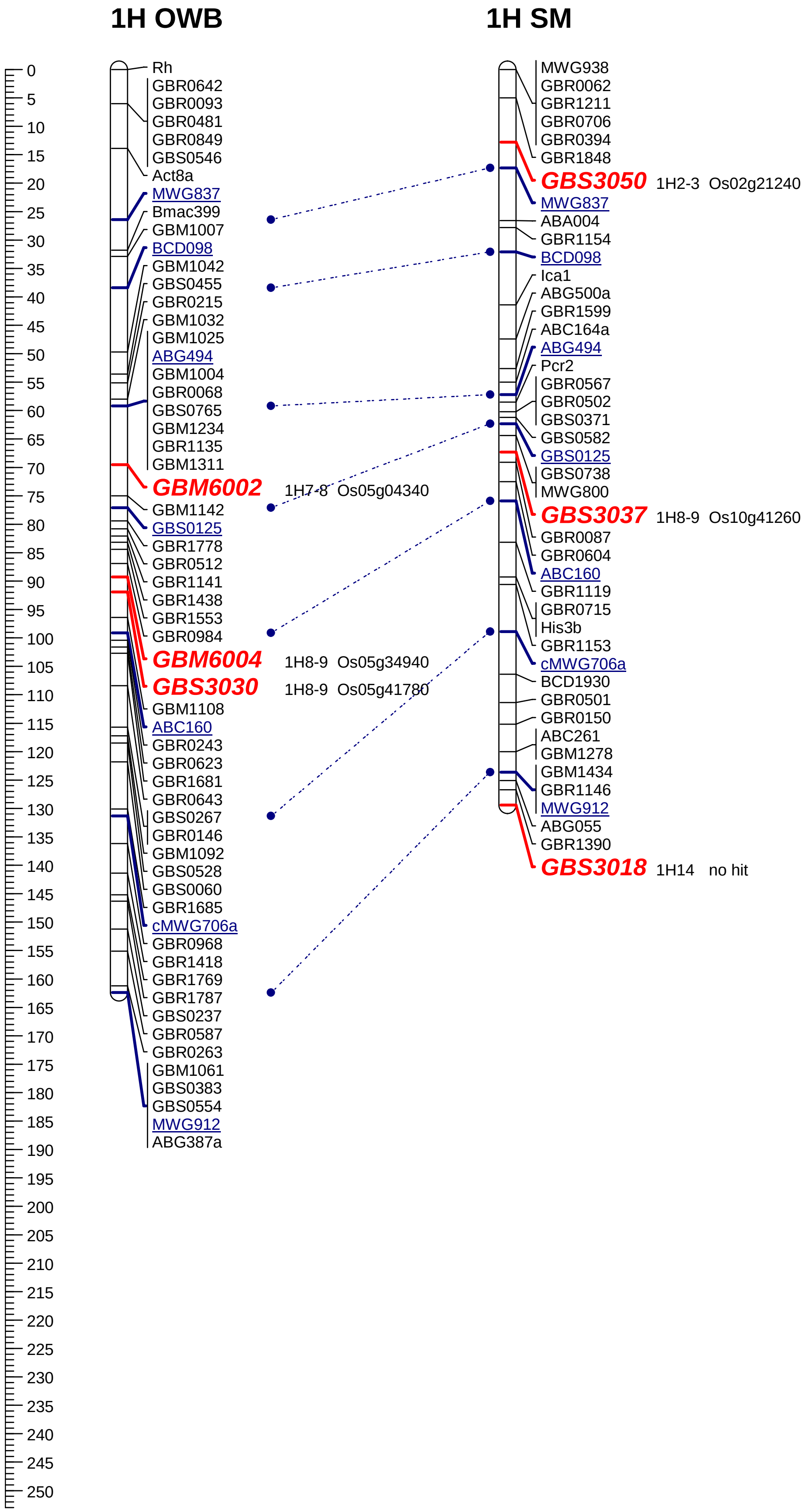
1H OWB
1H SM
0
5
10
15
20
25
30
35
40
45
50
55
60
65
70
75
80
85
90
95
100
105
110
115
120
125
130
135
140
145
150
155
160
165
170
175
180
185
190
195
200
205
210
215
220
225
230
235
240
245
250
Rh
GBR0642
GBR0093
GBR0481
GBR0849
GBS0546
Act8a
MWG837
Bmac399
GBM1007
BCD098
GBM1042
GBS0455
GBR0215
GBM1032
GBM1025
ABG494
GBM1004
GBR0068
GBS0765
GBM1234
GBR1135
GBM1311
GBM6002
1H7-8 Os05g04340
GBM1142
GBS0125
GBR1778
GBR0512
GBR1141
GBR1438
GBR1553
GBR0984
GBM6004
1H8-9 Os05g34940
GBS3030
1H8-9 Os05g41780
GBM1108
ABC160
GBR0243
GBR0623
GBR1681
GBR0643
GBS0267
GBR0146
GBM1092
GBS0528
GBS0060
GBR1685
cMWG706a
GBR0968
GBR1418
GBR1769
GBR1787
GBS0237
GBR0587
GBR0263
GBM1061
GBS0383
GBS0554
MWG912
ABG387a
MWG938
GBR0062
GBR1211
GBR0706
GBR0394
GBR1848
GBS3050
1H2-3 Os02g21240
MWG837
ABA004
GBR1154
BCD098
Ica1
ABG500a
GBR1599
ABC164a
ABG494
Pcr2
GBR0567
GBR0502
GBS0371
GBS0582
GBS0125
GBS0738
MWG800
GBS3037
1H8-9 Os10g41260
GBR0087
GBR0604
ABC160
GBR1119
GBR0715
His3b
GBR1153
cMWG706a
BCD1930
GBR0501
GBR0150
ABC261
GBM1278
GBM1434
GBR1146
MWG912
ABG055
GBR1390
GBS3018
1H14 no hit

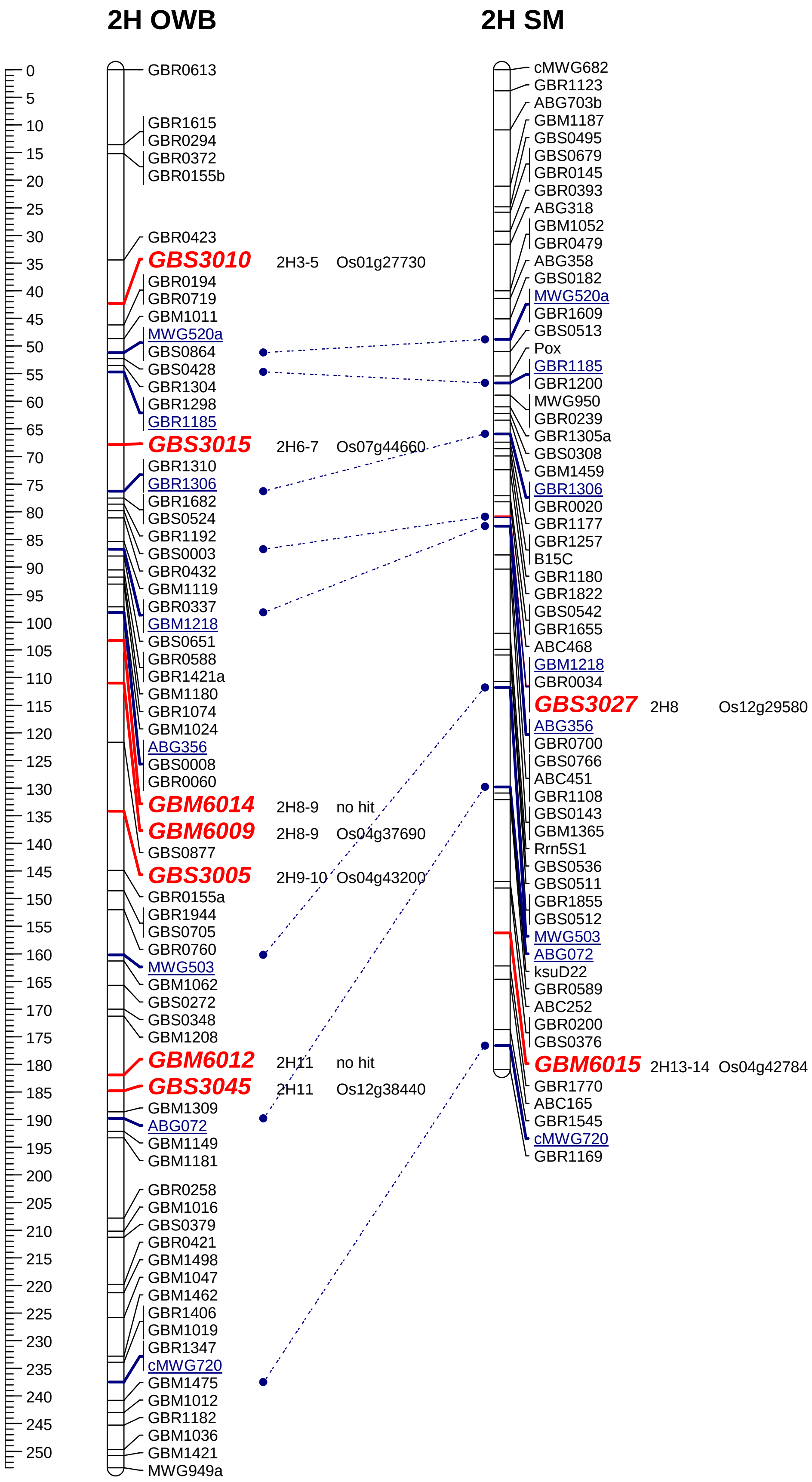

2H OWB
2H SM
0
5
10
15
20
25
30
35
40
45
50
55
60
65
70
75
80
85
90
95
100
105
110
115
120
125
130
135
140
145
150
155
160
165
170
175
180
185
190
195
200
205
210
215
220
225
230
235
240
245
250
GBR0613
GBR1615
GBR0294
GBR0372
GBR0155b
GBR0423
GBS3010
2H3-5
Os01g27730
GBR0194
GBR0719
GBM1011
MWG520a
GBS0864
GBS0428
GBR1304
GBR1298
GBR1185
GBS3015
2H6-7
Os07g44660
GBR1310
GBR1306
GBR1682
GBS0524
GBR1192
GBS0003
GBR0432
GBM1119
GBR0337
GBM1218
GBS0651
GBR0588
GBR1421a
GBM1180
GBR1074
GBM1024
ABG356
GBS0008
GBR0060
GBM6014
2H8-9
no hit
GBM6009
2H8-9
Os04g37690
GBS0877
GBS3005
2H9-10
Os04g43200
GBR0155a
GBR1944
GBS0705
GBR0760
MWG503
GBM1062
GBS0272
GBS0348
GBM1208
GBM6012
2H11
no hit
GBS3045
2H11
Os12g38440
GBM1309
ABG072
GBM1149
GBM1181
GBR0258
GBM1016
GBS0379
GBR0421
GBM1498
GBM1047
GBM1462
GBR1406
GBM1019
GBR1347
cMWG720
GBM1475
GBM1012
GBR1182
GBM1036
GBM1421
MWG949a
cMWG682
GBR1123
ABG703b
GBM1187
GBS0495
GBS0679
GBR0145
GBR0393
ABG318
GBM1052
GBR0479
ABG358
GBS0182
MWG520a
GBR1609
GBS0513
Pox
GBR1185
GBR1200
MWG950
GBR0239
GBR1305a
GBS0308
GBM1459
GBR1306
GBR0020
GBR1177
GBR1257
B15C
GBR1180
GBR1822
GBS0542
GBR1655
ABC468
GBM1218
GBR0034
GBS3027
2H8
Os12g29580
ABG356
GBR0700
GBS0766
ABC451
GBR1108
GBS0143
GBM1365
Rrn5S1
GBS0536
GBS0511
GBR1855
GBS0512
MWG503
ABG072
ksuD22
GBR0589
ABC252
GBR0200
GBS0376
GBM6015
2H13-14
Os04g42784
GBR1770
ABC165
GBR1545
cMWG720
GBR1169

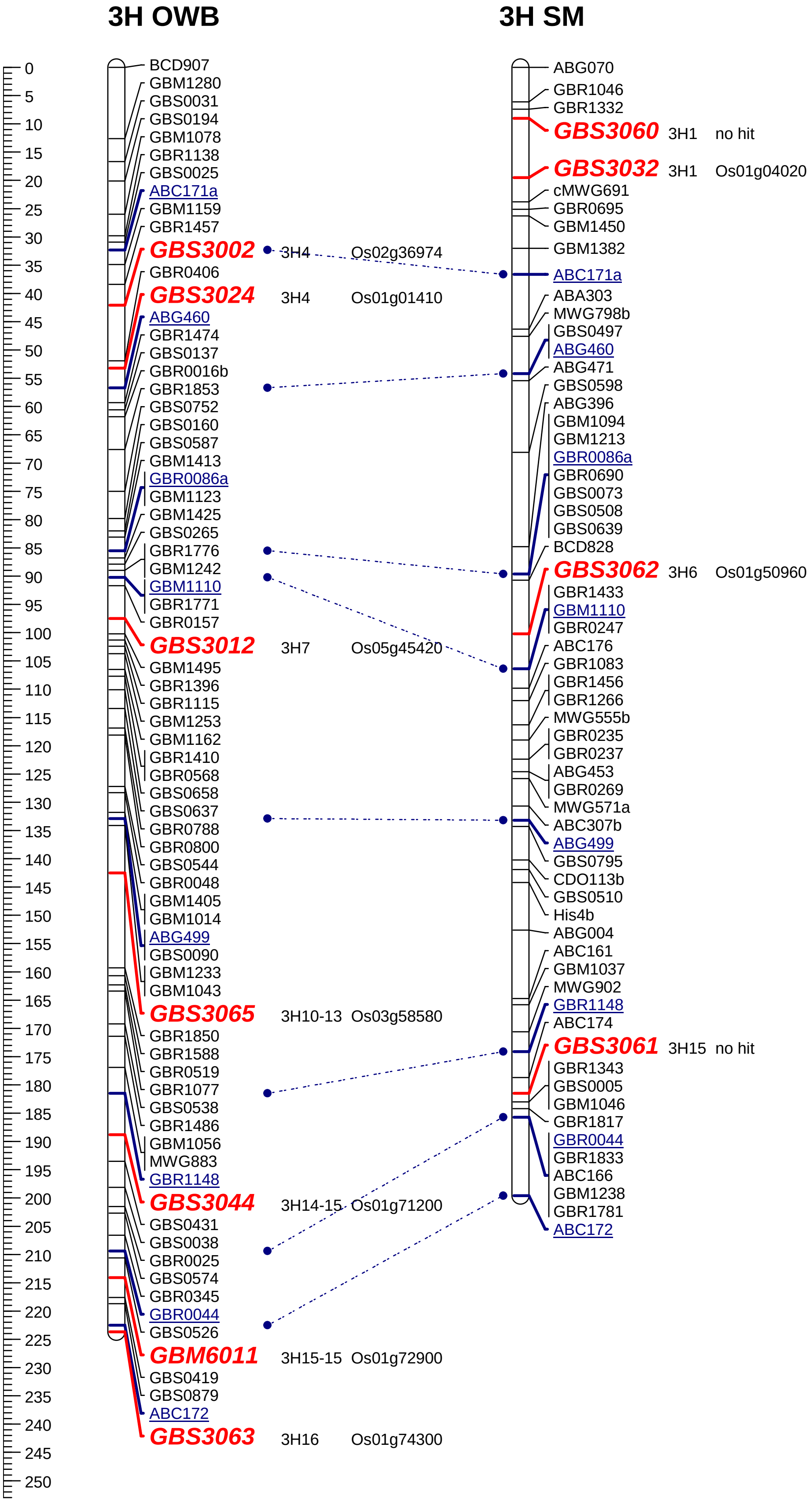
3H OWB
3H SM
BCD907
GBM1280
GBS0031
GBS0194
GBM1078
GBR1138
GBS0025
ABC171a
GBM1159
GBR1457
GBS3002 3H4 Os02g36974
GBR0406
GBS3024 3H4 Os01g01410
ABG460
GBR1474
GBS0137
GBR0016b
GBR1853
GBS0752
GBS0160
GBS0587
GBM1413
GBR0086a
GBM1123
GBM1425
GBS0265
GBR1776
GBM1242
GBM1110
GBR1771
GBR0157
GBS3012 3H7 Os05g45420
GBM1495
GBR1396
GBR1115
GBM1253
GBM1162
GBR1410
GBR0568
GBS0658
GBS0637
GBR0788
GBR0800
GBS0544
GBR0048
GBM1405
GBM1014
ABG499
GBS0090
GBM1233
GBM1043
GBS3065 3H10-13 Os03g58580
GBR1850
GBR1588
GBR0519
GBR1077
GBS0538
GBR1486
GBM1056
MWG883
GBR1148
GBS3044 3H14-15 Os01g71200
GBS0431
GBS0038
GBR0025
GBS0574
GBR0345
GBR0044
GBS0526
GBM6011 3H15-15 Os01g72900
GBS0419
GBS0879
ABC172
GBS3063 3H16 Os01g74300
ABG070
GBR1046
GBR1332
GBS3060 3H1 no hit
GBS3032 3H1 Os01g04020
cMWG691
GBR0695
GBM1450
GBM1382
ABC171a
ABA303
MWG798b
GBS0497
ABG460
ABG471
GBS0598
ABG396
GBM1094
GBM1213
GBR0086a
GBR0690
GBS0073
GBS0508
GBS0639
BCD828
GBS3062 3H6 Os01g50960
GBR1433
GBM1110
GBR0247
ABC176
GBR1083
GBR1456
GBR1266
MWG555b
GBR0235
GBR0237
ABG453
GBR0269
MWG571a
ABC307b
ABG499
GBS0795
CDO113b
GBS0510
His4b
ABG004
ABC161
GBM1037
MWG902
GBR1148
ABC174
GBS3061 3H15 no hit
GBR1343
GBS0005
GBM1046
GBR1817
GBR0044
GBR1833
ABC166
GBM1238
GBR1781
ABC172
0
5
10
15
20
25
30
35
40
45
50
55
60
65
70
75
80
85
90
95
100
105
110
115
120
125
130
135
140
145
150
155
160
165
170
175
180
185
190
195
200
205
210
215
220
225
230
235
240
245
250

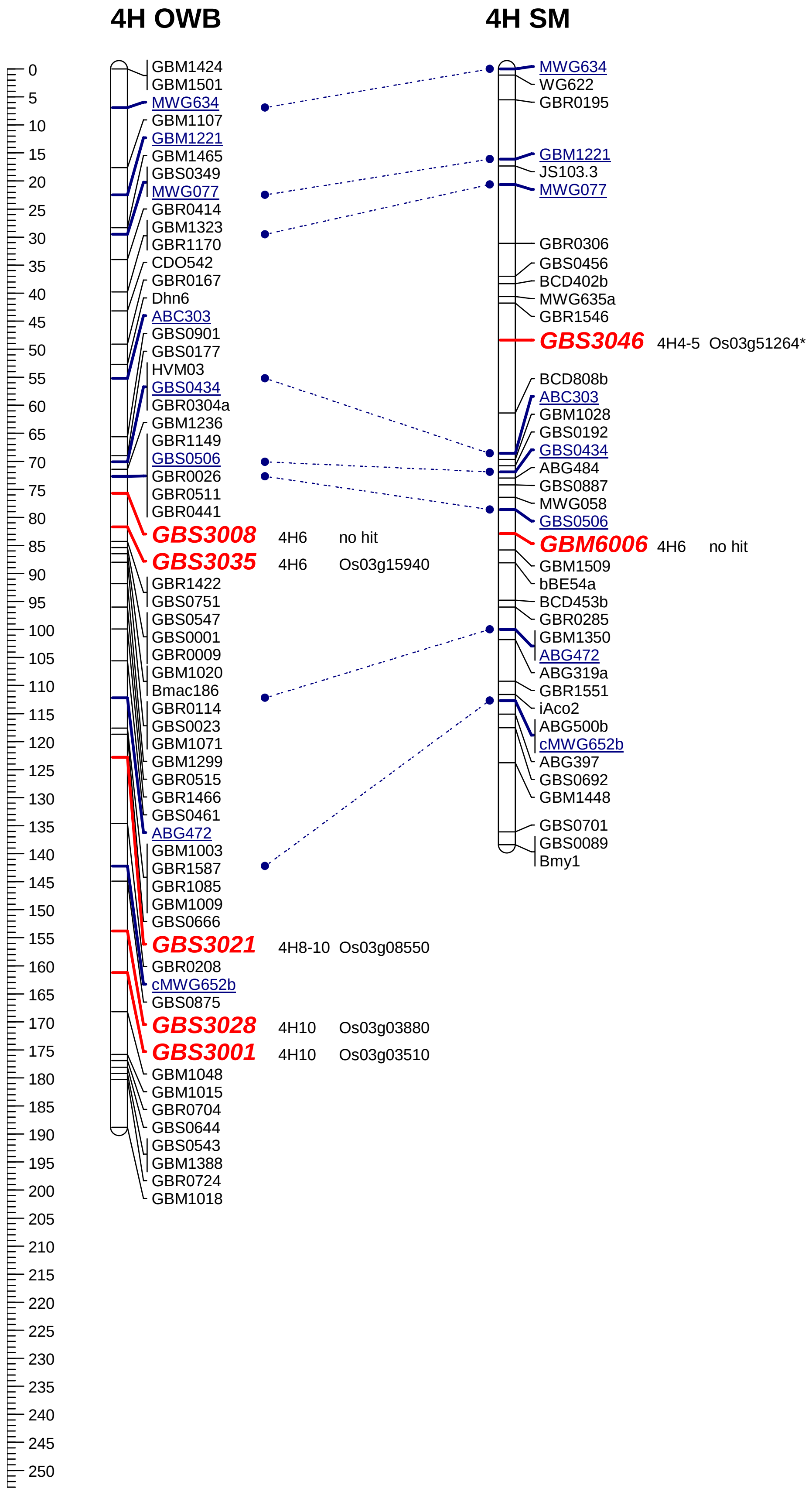
4H OWB
4H SM
GBM1424
GBM1501
MWG634
GBM1107
GBM1221
GBM1465
GBS0349
MWG077
GBR0414
GBM1323
GBR1170
CDO542
GBR0167
Dhn6
ABC303
GBS0901
GBS0177
HVM03
GBS0434
GBR0304a
GBM1236
GBR1149
GBS0506
GBR0026
GBR0511
GBR0441
GBS3008 4H6 no hit
GBS3035 4H6 Os03g15940
GBR1422
GBS0751
GBS0547
GBS0001
GBR0009
GBM1020
Bmac186
GBR0114
GBS0023
GBM1071
GBM1299
GBR0515
GBR1466
GBS0461
ABG472
GBM1003
GBR1587
GBR1085
GBM1009
GBS0666
GBS3021 4H8-10 Os03g08550
GBR0208
cMWG652b
GBS0875
GBS3028 4H10 Os03g03880
GBS3001 4H10 Os03g03510
GBM1048
GBM1015
GBR0704
GBS0644
GBS0543
GBM1388
GBR0724
GBM1018
MWG634
WG622
GBR0195
GBM1221
JS103.3
MWG077
GBR0306
GBS0456
BCD402b
MWG635a
GBR1546
GBS3046 4H4-5 Os03g51264*
BCD808b
ABC303
GBM1028
GBS0192
GBS0434
ABG484
GBS0887
MWG058
GBS0506
GBM6006 4H6 no hit
GBM1509
bBE54a
BCD453b
GBR0285
GBM1350
ABG472
ABG319a
GBR1551
iAco2
ABG500b
cMWG652b
ABG397
GBS0692
GBM1448
GBS0701
GBS0089
Bmy1

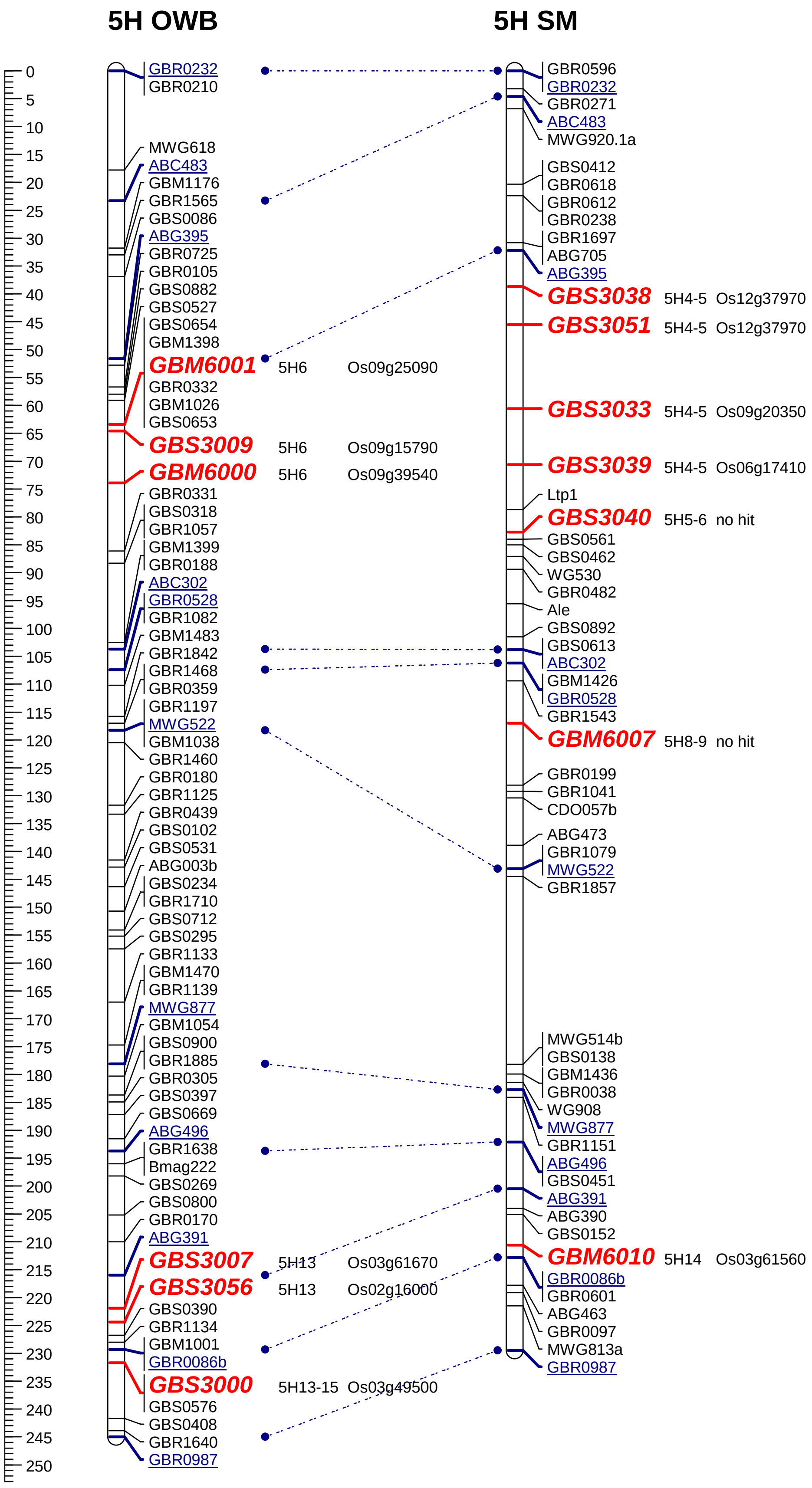

5H OWB
5H SM
0 5 10 15 20 25 30 35 40 45 50 55 60 65 70 75 80 85 90 95 100 105 110 115 120 125 130 135 140 145 150 155 160 165 170 175 180 185 190 195 200 205 210 215 220 225 230 235 240 245 250
GBR0232
GBR0210
MWG618
ABC483
GBM1176
GBR1565
GBS0086
ABG395
GBR0725
GBR0105
GBS0882
GBS0527
GBS0654
GBM1398
GBM6001 5H6 Os09g25090
GBR0332
GBM1026
GBS0653
GBS3009 5H6 Os09g15790
GBM6000 5H6 Os09g39540
GBR0331
GBS0318
GBR1057
GBM1399
GBR0188
ABC302
GBR0528
GBR1082
GBM1483
GBR1842
GBR1468
GBR0359
GBR1197
MWG522
GBM1038
GBR1460
GBR0180
GBR1125
GBR0439
GBS0102
GBS0531
ABG003b
GBS0234
GBR1710
GBS0712
GBS0295
GBR1133
GBM1470
GBR1139
MWG877
GBM1054
GBS0900
GBR1885
GBR0305
GBS0397
GBS0669
ABG496
GBR1638
Bmag222
GBS0269
GBS0800
GBR0170
ABG391
GBS3007 5H13 Os03g61670
GBS3056 5H13 Os02g16000
GBS0390
GBR1134
GBM1001
GBR0086b
GBS3000 5H13-15 Os03g49500
GBS0576
GBS0408
GBR1640
GBR0987
GBR0596
GBR0232
GBR0271
ABC483
MWG920.1a
GBS0412
GBR0618
GBR0612
GBR0238
GBR1697
ABG705
ABG395
GBS3038 5H4-5 Os12g37970
GBS3051 5H4-5 Os12g37970
GBS3033 5H4-5 Os09g20350
GBS3039 5H4-5 Os06g17410
Ltp1
GBS3040 5H5-6 no hit
GBS0561
GBS0462
WG530
GBR0482
Ale
GBS0892
GBS0613
ABC302
GBM1426
GBR0528
GBR1543
GBM6007 5H8-9 no hit
GBR0199
GBR1041
CDO057b
ABG473
GBR1079
MWG522
GBR1857
MWG514b
GBS0138
GBM1436
GBR0038
WG908
MWG877
GBR1151
ABG496
GBS0451
ABG391
ABG390
GBS0152
GBM6010 5H14 Os03g61560
GBR0086b
GBR0601
ABG463
GBR0097
MWG813a
GBR0987

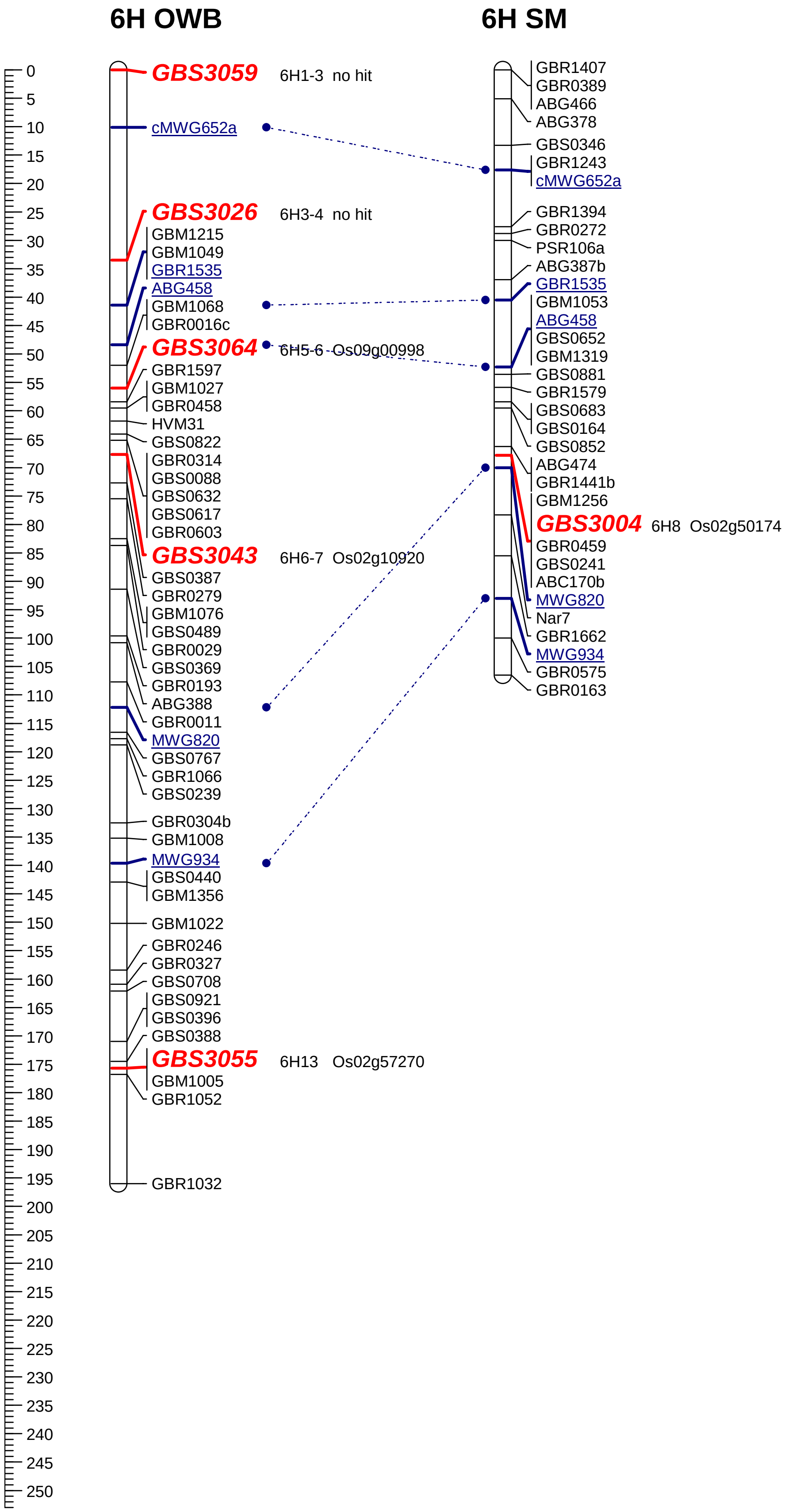

6H OWB
6H SM
0
5
10
15
20
25
30
35
40
45
50
55
60
65
70
75
80
85
90
95
100
105
110
115
120
125
130
135
140
145
150
155
160
165
170
175
180
185
190
195
200
205
210
215
220
225
230
235
240
245
250
GBS3059 6H1-3 no hit
cMWG652a
GBS3026 6H3-4 no hit
GBM1215
GBM1049
GBR1535
ABG458
GBM1068
GBR0016c
GBS3064 6H5-6 Os09g00998
GBR1597
GBM1027
GBR0458
HVM31
GBS0822
GBR0314
GBS0088
GBS0632
GBS0617
GBR0603
GBS3043 6H6-7 Os02g10920
GBS0387
GBR0279
GBM1076
GBS0489
GBR0029
GBS0369
GBR0193
ABG388
GBR0011
MWG820
GBS0767
GBR1066
GBS0239
GBR0304b
GBM1008
MWG934
GBS0440
GBM1356
GBM1022
GBR0246
GBR0327
GBS0708
GBS0921
GBS0396
GBS0388
GBS3055 6H13 Os02g57270
GBM1005
GBR1052
GBR1032
GBR1407
GBR0389
ABG466
ABG378
GBS0346
GBR1243
cMWG652a
GBR1394
GBR0272
PSR106a
ABG387b
GBR1535
GBM1053
ABG458
GBS0652
GBM1319
GBS0881
GBR1579
GBS0683
GBS0164
GBS0852
ABG474
GBR1441b
GBM1256
GBS3004 6H8 Os02g50174
GBR0459
GBS0241
ABC170b
MWG820
Nar7
GBR1662
MWG934
GBR0575
GBR0163

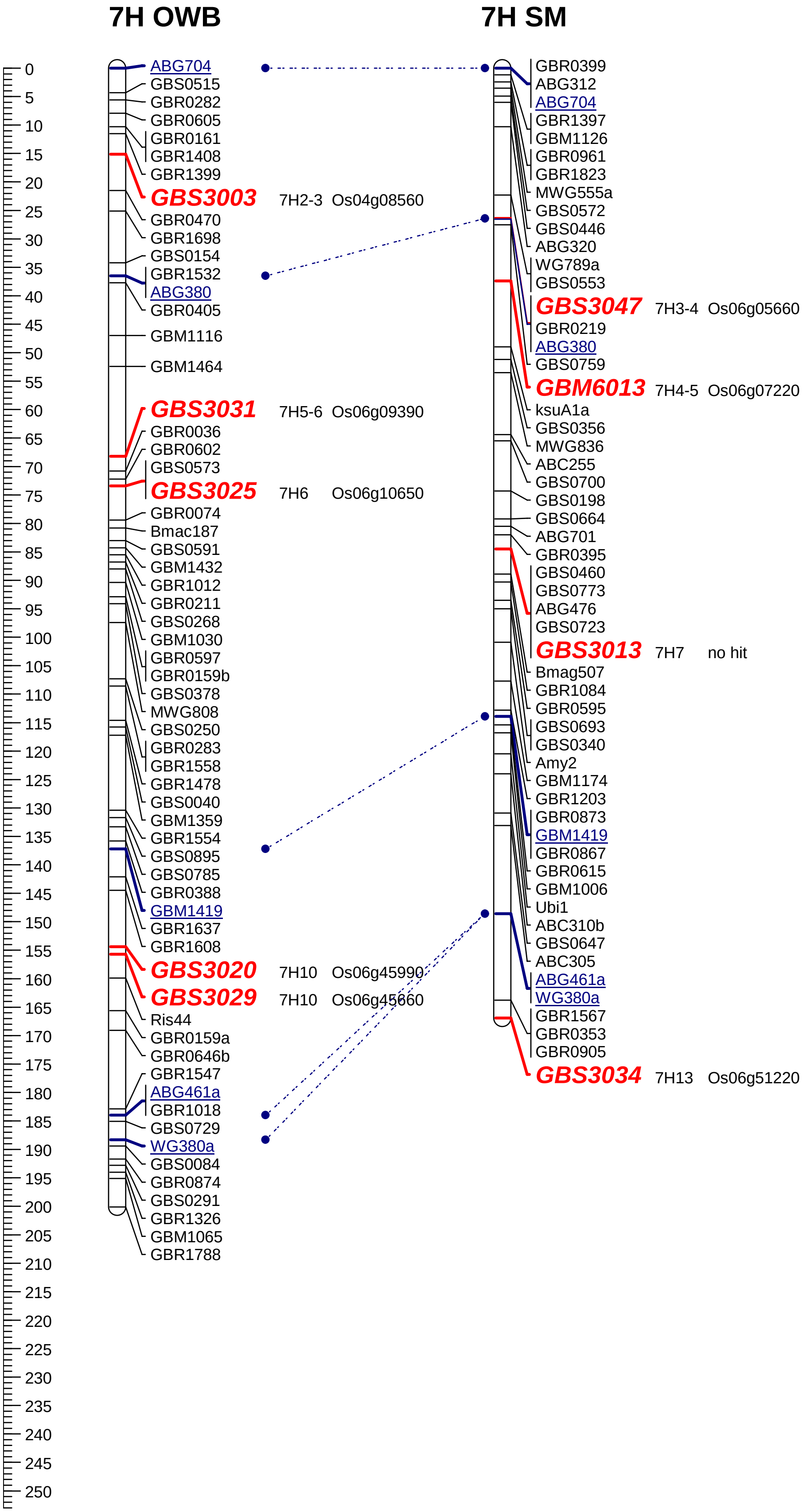
7H OWB
7H SM
0
5
10
15
20
25
30
35
40
45
50
55
60
65
70
75
80
85
90
95
100
105
110
115
120
125
130
135
140
145
150
155
160
165
170
175
180
185
190
195
200
205
210
215
220
225
230
235
240
245
250
ABG704
GBS0515
GBR0282
GBR0605
GBR0161
GBR1408
GBR1399
GBS3003 7H2-3 Os04g08560
GBR0470
GBR1698
GBS0154
GBR1532
ABG380
GBR0405
GBM1116
GBM1464
GBS3031 7H5-6 Os06g09390
GBR0036
GBR0602
GBS0573
GBS3025 7H6 Os06g10650
GBR0074
Bmac187
GBS0591
GBM1432
GBR1012
GBR0211
GBS0268
GBM1030
GBR0597
GBR0159b
GBS0378
MWG808
GBS0250
GBR0283
GBR1558
GBR1478
GBS0040
GBM1359
GBR1554
GBS0895
GBS0785
GBR0388
GBM1419
GBR1637
GBR1608
GBS3020 7H10 Os06g45990
GBS3029 7H10 Os06g45660
Ris44
GBR0159a
GBR0646b
GBR1547
ABG461a
GBR1018
GBS0729
WG380a
GBS0084
GBR0874
GBS0291
GBR1326
GBM1065
GBR1788
GBR0399
ABG312
ABG704
GBR1397
GBM1126
GBR0961
GBR1823
MWG555a
GBS0572
GBS0446
ABG320
WG789a
GBS0553
GBS3047 7H3-4 Os06g05660
GBR0219
ABG380
GBS0759
GBM6013 7H4-5 Os06g07220
ksuA1a
GBS0356
MWG836
ABC255
GBS0700
GBS0198
GBS0664
ABG701
GBR0395
GBS0460
GBS0773
ABG476
GBS0723
GBS3013 7H7 no hit
Bmag507
GBR1084
GBR0595
GBS0693
GBS0340
Amy2
GBM1174
GBR1203
GBR0873
GBM1419
GBR0867
GBR0615
GBM1006
Ubi1
ABC310b
GBS0647
ABC305
ABG461a
WG380a
GBR1567
GBR0353
GBR0905
GBS3034 7H13 Os06g51220
